# Supplementary material for: Revealing Fermi surface evolution and Berry curvature in an ideal type-II Weyl semimetal
Source: Nat Commun. 2024 Mar 14;15:2310. doi: 10.1038/s41467-024-46633-w (PMC10940624; doi:10.1038/s41467-024-46633-w)
Supplement: Supplementary file 1 — Supplementary Information [file 41467_2024_46633_MOESM1_ESM.pdf]

Supplementary Information for: "Revealing Fermi Surface Evolution and Berry Curvature in an Ideal Type-II Weyl Semimetal"

Qianni Jiang[1], Johanna Palmstrom[2], John Singleton[2], Shalinee Chikara[3], David Graf[3], Chong Wang[4], Yue Shi[4], Paul Malinowski[1], Aaron Wang[1], Zhong Lin[1], Lingnan Shen[1], Xiaodong Xu[1,4], Di Xiao[4,1], and Jiun-Haw Chu[1]

[1]Department of Physics, University of Washington, Seattle, WA 98105, USA
[2]National High Magnetic Field Laboratory, Los Alamos National Laboratory, Los Alamos, NM 87545, USA
[3]National High Magnetic Field Laboratory, Florida State University, Tallahassee, FL 32306, USA
[4]Department of Material Science and Engineering, University of Washington, Seattle, WA 98105, USA

I. Two-band Hall Fitting

We fitted the multiband Hall resistivity data near the Weyl nodes as shown in Fig. 4(c) in the main text to the effective two-band model with the diagonal resistivity at the upper critical field H_{c2} as a constraint.

$$\rho_{xy} = \frac{1}{e}\frac{(n_h\mu_h^2 - n_e\mu_e^2) + \mu_h^2\mu_e^2B^2(n_h - n_e)}{(n_h\mu_h + n_e\mu_e)^2 + \mu_h^2\mu_e^2B^2(n_h - n_e)^2}B \quad (1)$$

$$\rho_{xx}(B = H_{c2}) = \frac{1}{e}\frac{(n_h\mu_h + n_e\mu_e) + (n_e\mu_e\mu_h^2 + n_h\mu_h\mu_e^2)B^2}{(n_h\mu_h + n_e\mu_e)^2 + \mu_h^2\mu_e^2B^2(n_h - n_e)^2} \quad (2)$$

where n_e (n_h) is the electron (hole) carrier density, and μ_e (μ_h) is the electron (hole) mobility. The fitting as shown in Supplementary Fig. 1(a) yields electron density 2.37×10^{17} cm^{-3} with a mobility of 1100 cm^{2}V^{-1}s^{-1} and hole density 2.35×10^{17} cm^{-3} with a mobility of 1300 cm^{2}V^{-1}s^{-1} at the base. As the temperature increases, the multi-band Hall properties persist, yet with a deviation from the base-temperature Hall resistivity (Supplementary Fig. 1(b)). This was also captured by the two-band Hall fits. A sudden drop in carrier mobilities and a sudden increase in carrier densities are noticed as the temperature increases and reaches the ground state magnetic ordering temperature (T_N) near 24 K, suggesting a possible modification of the electronic structure caused by the magnetic exchange interactions.

It is worth noting that in Fig. 3 (e), the carrier type associated with the two oscillation frequencies observed for the x = 0.7 sample is determined by comparing the carrier densities extracted from the oscillation frequencies assuming the Fermi pockets are isotropic and the carrier densities from the two-band Hall fitting. However, it is also possible that the higher frequency orbit corresponds to a hole pocket and the lower one corresponds to electron pockets if the Fermi pockets are highly anisotropic. In both scenarios, the sample with x = 0.7 exhibits the coexistence of electron and hole pockets.

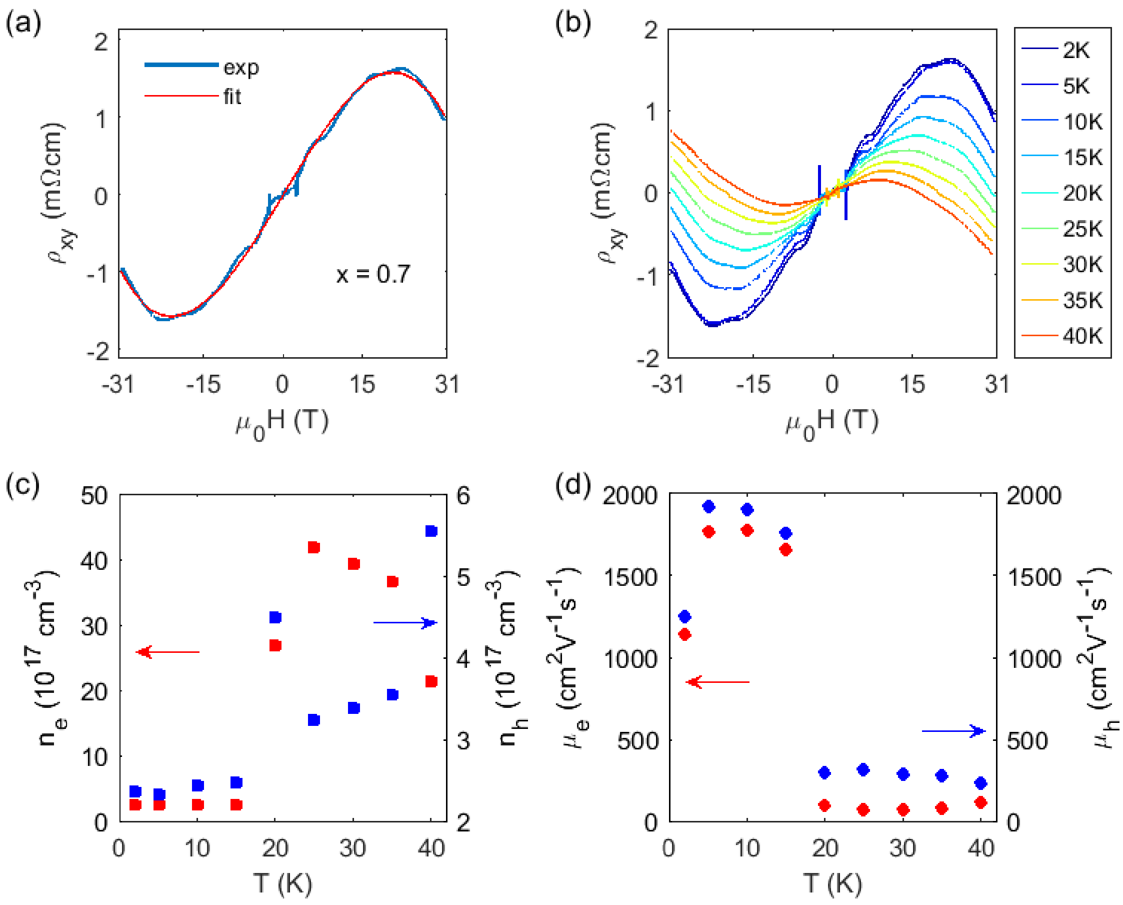

Supplementary Fig. 1. Multiband Hall resistivity of $MnBi_{1.3}Sb_{0.7}Te_4$ (a) Hall resistivity (blue) and the fit to the two-band model (red) with $\mu_0\mathbf{H}$//[001] at 2K (b) The field dependence of Hall resistivity at various temperatures with $\mu_0\mathbf{H}$//[001] (c)-(d) The temperature dependence of the fitting parameters of the effective two-band model: (c) electron carrier density (red) and hole carrier density (blue) (d) electron mobility (red) and hole mobility (blue).

II. Analytical fitting to the Lifshitz-Kosevich formula

The multiple frequencies of quantum oscillations present in $MnBi_{2-x}Sb_xTe_4$ are difficult to resolve. One reason is because of the low oscillation frequencies (< 100 T) and the limited field range (the oscillation can only be seen above the spin-flip transition at 7 T.) Another difficulty is caused by the complex shape of the background magnetoresistance which can mix with the oscillation at high fields. To overcome these difficulties, we fit the oscillation signal analytically to the Lifshitz Kosevich (LK) formula assuming two frequencies and a polynomial background. The LK formula for the fundamental oscillations (without higher harmonics) of a single sheet of Fermi surface is given by:

$$\frac{\Delta\rho_{xx}}{\rho_{xx}} \propto R_T R_D \times \cos[2\pi(\frac{F_S}{B} + \Gamma)] \quad (3)$$

where F_S is the frequency of the oscillation, Γ is the phase factor, R_T and R_D are the thermal damping factor and Dingle temperature factor, respectively. The thermal damping factor R_T is determined by temperature T and effective mass m^*.

$$R_T = \frac{\alpha T m^*}{B} \sinh\left(\frac{\alpha T m^*}{B}\right) \tag{4}$$

where is α a constant $\frac{2\pi^2 k_B m_e}{e\hbar} \approx 14.69\ T/K$. At base temp, the thermal damping factor R_T is very close to 1. The Dingle temperature factor R_D describes the reduction of the oscillation amplitude caused by electron scattering.

$$R_D = exp\left(-\frac{\alpha T_D m^*}{B}\right) \tag{5}$$

T_D is the Dingle temperature, given by $\hbar/2\pi k_B \tau_s$, where τ_s is the scattering time. In the case of two coexisting Fermi-surface sections. or two band extrema within a single Fermi-surface section, as illustrated in Fig. 1(a) in the main text, one needs to fit an analytical function containing two contributions from Eq. (1), plus a polynomial background.

$$\rho_{xx} = a \times \exp\left(-\frac{b}{B}\right) \times \cos\left[2\pi\left(\frac{F_{S1}}{B} + \Gamma_1\right)\right] + c \times \exp\left(-\frac{d}{B}\right) \times \cos\left[2\pi\left(\frac{F_{S2}}{B} + \Gamma_2\right)\right] + \rho_{xx,\,bg}(B) \tag{6}$$

where *a, b, c, d*, F_{S1}, F_{S2}, Γ_1 and Γ_2 are fitting parameters, and $\rho_{xx,\,bg}(\boldsymbol{B})$ is the polynomial background. Simultaneously fitting the oscillatory signals and magnetoresistance background enables the background to be modulated by the oscillations when needed, and vice versa. Such a fit has a large parameter space. Therefore, it is important to find a good starting point for each parameter to make the fit converge to the correct solution. In the specific case of the quantum oscillations of FM $MnBi_{2-x}Sb_xTe_4$, we find that one oscillation signal associated with a lower frequency always damps faster than the other, which might be the reason that only one frequency of the Shubnikov-de Haas (SdH) oscillations was observed at low magnetic fields in previous studies. Given this knowledge, we first subtract a polynomial background at low field and fit the major frequency with a single-frequency LK formula to obtain initial values of the fit parameters. We then conducted a grid search over decades of different F_{S2} and damping factor d to search for the best fit with the lowest residual sum of squares.

Supplementary Fig. 2 shows an example of the analytical fitting of the SdH oscillations to the single-frequency LK formula. Supplementary Fig. 2(a) shows the fit to the single frequency LK formular at low field (< 22 T), while Supplementary Fig. 2(b) displays the fit to the LK formula with two oscillation frequencies and the magnetoresistance background. The beating effect and the large upturn in the oscillatory signals at high magnetic field are well captured by the analytical fit. However, a small deviation from the data was observed in fitting at a lower magnetic field, which might be related to the anomalous oscillation shift caused by magnetization softening as discussed in previous reports[1].

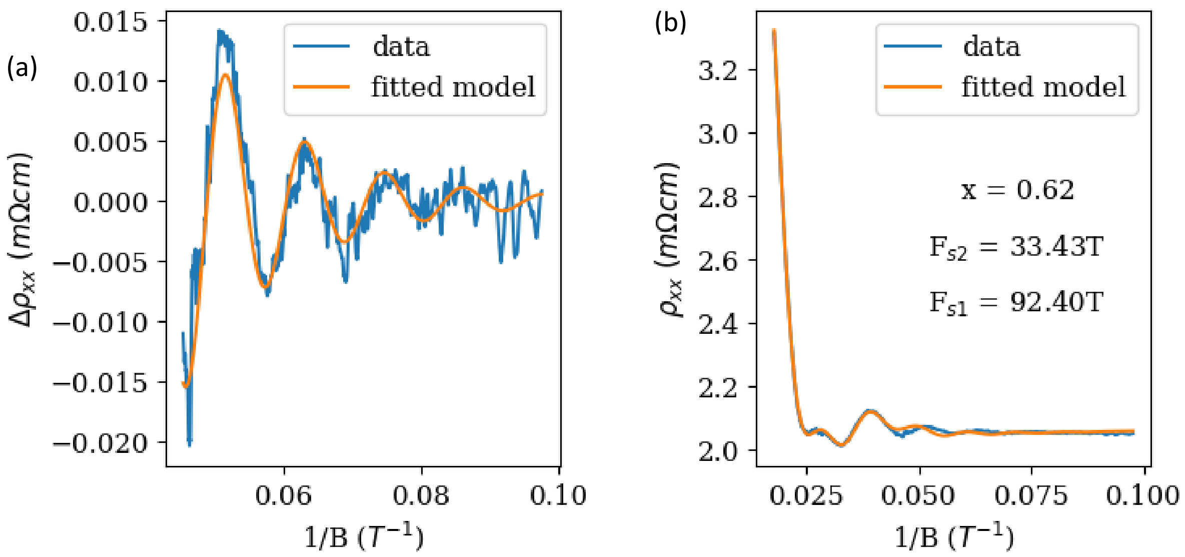

Supplementary Fig. 2 Analytical fitting of SdH oscillations to the Lifshitz Kosevich formula (a) analytical fit to the single frequency LK formula (Eq. (3)) at low field (< 22T) (b) analytical fit to the LK formula with two oscillation frequencies and magnetoresistance background (Eq. (6)).

III. Anomalous Hall Effect

Supplementary Fig. 3 (a) displays the raw data depicting the Hall resistivity ρ_{xy} versus magnetic field μ_0H for samples with varying carrier densities (color-coded). To extract the anomalous Hall resistivity ρ_{xy}^A for each sample, we fit the linear slope above the upper critical field H_{C2} and subtract this linear component from the Hall resistivity, $\rho_{xy}^A = \rho_{xy} - R_{H,high}\mu_0 H$, as illustrated in Supplementary Fig. 3(b). Subsequently, we approximately convert the anomalous Hall resistivity ρ_{xy}^A to anomalous Hall conductivity σ_{xy}^A by utilizing both longitudinal ρ_{xx} and Hall resistivities ρ_{xy} according to the formula $\sigma_{xy}^A = \frac{-\rho_{xy}^A}{\rho_{xx}^2+\rho_{xy}^2}$, as summarized in Supplementary Fig. 3 (c). Finally, we plot the anomalous Hall conductivity as a function of the carrier densities extracted from the low field (< $\mathbf{H}_{c1}$) Hall resistivity as Fig. 5(b) in the main text demonstrates.

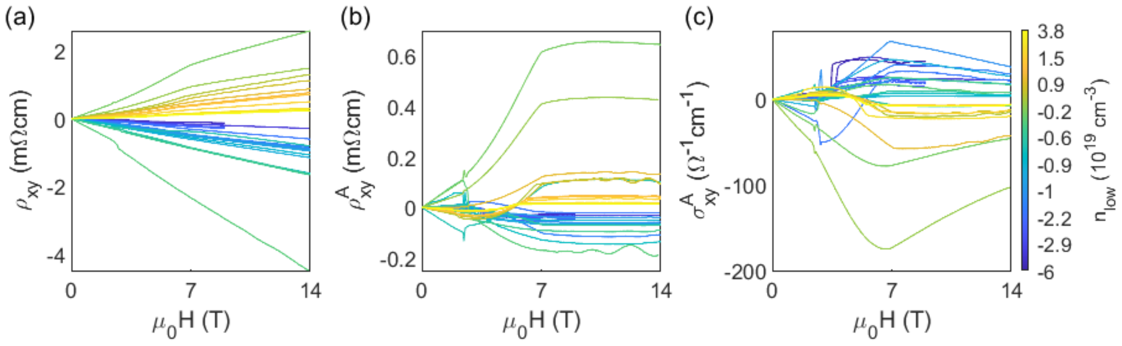

Supplementary Fig. 3 Anomalous Hall effect of $MnBi_{2-x}Sb_xTe_4$ (a) Hall resistivity ρ_{xy} as a function of magnetic field $\mu_0\mathbf{H}$. Samples with varying carrier densities are color coded. Carrier densities n_{low} are estimated by fitting the linear slope under a low magnetic field (< $\mathbf{H}_{c1}$) in the antiferromagnetic state. (b) Anomalous Hall resistivity ρ_{xy}^{A} as a function of magnetic field μ_0H obtained by subtracting a linear contribution fitted under high magnetic fields (> $\mathbf{H}_{c2}$) from the Hall resistivity in panel (a). (c) Anomalous Hall conductivity σ_{xy}^{A} as a function of magnetic field $\mu_0\mathbf{H}$ for samples with varying carrier densities.

References

1 Jiang, Q. *et al.* Quantum oscillations in the field-induced ferromagnetic state of $MnBi_{2-x}Sb_xTe_4$. *Physical Review B* **103**, 205111, doi:10.1103/PhysRevB.103.205111 (2021).
